# The Effect of Probiotics Supplementation on *Helicobacter pylori* Eradication Rates and Side Effects during Eradication Therapy: A Meta-Analysis

**DOI:** 10.1371/journal.pone.0111030

**Published:** 2014-11-03

**Authors:** Yini Dang, Jan D. Reinhardt, Xiaoying Zhou, Guoxin Zhang

**Affiliations:** 1 Department of Gastroenterology, The First Affiliated Hospital of Nanjing Medical University, Nanjing, China, and First Clinical Medical College of Nanjing Medical University, Nanjing, China; 2 Institute for Disaster Management and Reconstruction, Sichuan University, Chengdu, China, and Hong Kong Polytechnical University, Hung Hom, Hong Kong, China; 3 Department of Health Sciences, University of Lucerne, Lucerne, Switzerland; 4 Swiss Paraplegic Research, Nottwil, Switzerland; Fundació Institut d'Investigació en Ciències de la Salut Germans Trias i Pujol. Universitat Autònoma de Barcelona. CIBERES, Spain

## Abstract

**Background:**

Previous meta-analyses reported that probiotics improve the effectiveness of *Helicobacter pylori* (*H. pylori*) eradication during antibiotic therapy, while results regarding a possible reduction of side effects remained inconclusive. Moreover, the effectiveness of different strains of probiotics has not been studied so far. It is further conceivable that probiotics will produce additional effects only if antibiotics are relatively ineffective.

**Methods:**

This meta-analysis includes eligible randomized controlled trials examining effects of probiotics supplementation on eradication rates (ER) and side effects, published up to May 2014. Sub-group analysis was performed to compare different probiotic strains and antibiotic therapies with different effectiveness in controls (ER <80% vs.>80%). Publication bias was assessed with funnel plots and Harbord's test. The quality of the trials was assessed with the Cochrane risk of bias tool.

**Results:**

Thirty-three RCTs involving a total of 4459 patients met the inclusion criteria in case of eradication rates of which 20 assessed total side effects in addition. Overall, the pooled eradication rate in probiotics supplementation groups was significantly higher than in controls (ITT analysis: RR 1.122, 95% CI 1.086–1.159, PP analysis: RR 1.114, 95% CI 1.070–1.159). Sub group-analysis could, however, confirm this finding only for four individual strains (*Lactobacillus acidophilus*, *Lactobacillus casei* DN-114001, *Lactobacillus gasseri*, and *Bifidobacterium infantis* 2036) and for relatively ineffective antibiotic therapies. There was a significant difference between groups in the overall incidence of side effects (RR 0.735, 95% CI 0.598–0.902). This result was, however, only confirmed for non-blinded trials.

**Conclusions:**

The pooled data suggest that supplementation with specific strains of probiotics compared with eradication therapy may be considered an option for increasing eradication rates, particularly when antibiotic therapies are relatively ineffective. The impact on side effects remains unclear and more high quality trials on specific probiotic strains and side effects are thus needed.

## Introduction

*‘Helicobacter pylori* (*H. pylori*) is a common bacterium infecting about half of the world's population. It is causally associated with a diverse spectrum of gastrointestinal disorders' [1]. Eradication of *H. pylori* is necessary for the management of *H. pylori*-related complications. The recommended first approach for *H. pylori* eradication is standard triple antibiotic therapy. Other choices include sequential therapy and quadruple therapy [2]. However, due to antibiotic resistance and patient non-compliance, several studies showed widespread failure of antibiotic therapy [3], [4]. Driven by the growing necessity for alternative solutions to eradication regimens, some studies have started to focus on probiotics [5], i.e. ‘live microorganisms which when administered in adequate amounts confer a health benefit on the host' [6]. The most commonly used probiotic bacteria belong to the genera *Lactobacillus* and *Bifidobacterium*, and these also include several yeasts such as *Saccharomyces boulardii* [7]. It has been hypothesized that probiotics could improve *H. pylori* eradication and reduce side effects during therapy.

Although seven meta-analyses [8]–[14] on this topic have been conducted over the past seven years, we perform an additional meta-analysis based on the following considerations: 1) The number of articles included in six of the seven previously conducted meta-analysis was ten or less [9]–[14]. In 2010–2014, many new studies were performed to evaluate the effectiveness of probiotics supplementation increasing the evidence base. 2) There are various genera of probiotics used in clinical practice and most meta-analyses [9], [11], [12], [13] solely concentrated on one specific strain. 3) Opposite conclusions were drawn regarding side effects: Sachdeva [10], Zou [9], and Zheng [13] report no significant reduction of overall side effects in probiotics groups. However, Tong [8], Szajewska [11], Wang [12], and Li [14] arrive at opposite conclusions. Moreover, no sensitivity analysis regarding side effects has been conducted so far. 4) While all meta-analysis conducted so far reported increased eradication rates due to probiotics supplementation, no sensitivity analysis with regard to the effectiveness of the antibiotic therapy has been conducted, i.e. it is conceivable that probiotics supplementation will solely increase effectiveness in relatively ineffective antibiotic regimens.

This meta-analysis is thus designed to evaluate the current evidence regarding effects of probiotics supplementation compared with eradication triple therapy only on H. pylori eradication rates and side effects. Moreover, we aim to compare different strains of probiotics as well as differentially effective antibiotic therapies and to evaluate the quality of the trials conducted so far.

## Methods

### Search Strategy

We performed a literature search in Pubmed and Web of Science, covering papers published up to May 2014. A combination of the following keywords was used: (probiotics OR prebiotics OR *Bifidobacterium* OR *Lactobacillus* OR *Saccharomyces*) AND (*Helicobacter pylori* OR *H. pylori*). The reference lists of the selected papers and the seven previous meta-analyses were also screened for other eligible articles that may have been missed in the initial search. Next we scanned the titles and abstracts of the trials identified in the computerized search to exclude studies that were obviously irrelevant and scrutinized the full-texts of the remaining studies.

### Inclusion and Exclusion Criteria

The following criteria were used for the selection of relevant articles: 1) studies should be randomized controlled trials (RCTs); 2) study populations should never have been treated for *H. pylori* infection before; 3) studies should include at least two branches of treatment consisting of (a) studies should have patients in a control group who received antibiotic therapy, (b) there should be patients in an intervention group who received probiotics plus the identical antibiotic therapy; 4) there should be data on successful eradication rates available; 5) for the analysis of side effects data on occurrence of side effects during treatment were required as well. Exclusion criteria were as follows: 1) the design and the definition of the trials were obviously different from those specified above; 2) essential information was not provided; 3) papers were letters, commentaries, editorials, reviews and duplicate publications; 4) articles were written in a language other than English.

### Data Extraction

Two authors extracted data independently from all eligible papers. For conflicting evaluations, another author was consulted to solve the dispute and a final decision was made by the majority of the votes. The data extracted included authors, year of publication, base characteristics of the patients, details of the *H. pylori* eradication therapy, details related to interventions, primary and secondary outcomes, and confirmation methods of *H. pylori* infection. Extracted information was entered into a database.

### Assessment of Study Quality

We used the Cochrane Tool of Bias [15] to assess study quality. Two authors independently evaluated all studies. Results were then compared and discussed to form consensus. If consensus could not be reached, another author was consulted and a decision made by the majority of the votes.

### Statistical Analysis

Statistical heterogeneity was analyzed with Chi-squared distributed Chochran's Q and the I-squared statistics (I^2^ = 100%× (Q-df)/Q). I-square indicates the percentage of variation across studies due to hetereogeneity as opposed to chance. We assumed sufficient homogeneity to apply a fixed-effects model (Mantel-Haenszel) for the meta-analysis if I-square was under 40% and/or Q was not significant at p<0.05. Otherwise, we opted for a random-effects model (DerSimonian and Laird). The jacknife was used to assess the influence of individual studies, i.e. estimation of the overall effect was repeated while omitting one study at each time. Funnel plots and Harbord's modified test for small study effects were used to assess publication bias [16]. The influence of the probiotic strain applied (multi-strain interventions were excluded from this analysis), blinded vs. non-blinded trials, and in the case of eradication rates, the effectiveness of antibiotic therapy in the control group (eradication rate >80% vs. <80%) was assessed with sub-group analysis. Concerning eradication rates, meta-regression was used to determine the influence of studies in pediatric vs. adult as well as symptomatic and asymptomatic populations. All statistical analysis was performed with STATA 12.0 (Stata Corporation, Texas, USA).

## Results

### Study Characteristics

**Figure 1:**
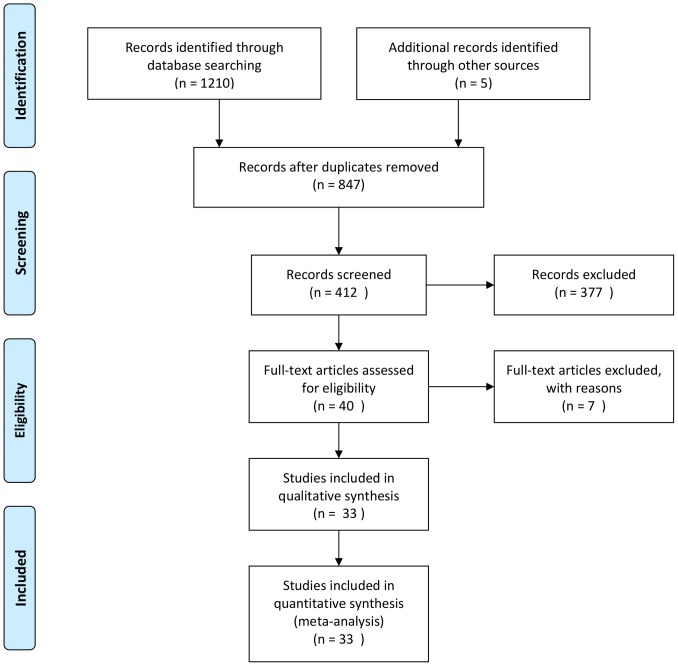
Flow of article selection.

The bibliographic search yielded 1114 articles, 40 of which were reviewed in full text (Figure 1) [17]–[56]. Of these studies, 33 RCTs [17]–[49] met the inclusion criteria for the analysis of eradication rates and 20 were eligible for analyzing side effects. These trials randomized a total of 4459 patients, 4261 of which were followed up. Nine studies enrolled only children [24]–[26], [33], [34], [36], [44], [45], [49] and 24 were undertaken exclusively with adults [17]–[23], [27]–[32], [35], [37]–[43], [46]–[48]. From all the included trials, 29 used PPI-triple therapy [17]–[25], [27]–[29], [31]–[41], [43]–[46], [48], [49], three used sequential therapy [26], [30], [42] and one used bismuth-quadruple therapy [47]. These studies were undertaken in Argentina [25], Brazil [46], mainland China [41], [49], China (Taiwan) [21], Finland [23], France [38], Iran [43], [45], [47], Italy [17]–[20], [22], [26], [29], [30], [32], [42], [44], Japan [40], Korea [31], [35], Poland [27], [34], Romania [33], Turkey [28], [36], [37], [39], United Kingdom [24] and the United Arabian Emirates [48].(Table 1)

**Table 1:** Characteristica of studies included in the meta-analysis.

Authors (references)	Year	Area	Case Number (treatment/control)	Patients	Diagnositic Methods	Eradication Therapy	Probiotics Supplementation	% Eradication of Treatment (no. of patients)	% Rradication of Control (no. of patients)	% Side Effects (no. of patients) (treatment/control)
Canducci [15]	2000	Italy	120 (60/60)	symptomatic adults	UBT +Histology/UBT-Histology (6 weeks later)	rabeprazole 20 mg bid, clarithromycin 250 mg tid, amoxicillin 500 mg tid, 7 days	Lacteol Fort (Bruschettini s.r.l., Genoa, Italy), containing 5×10∧9 heat-killed *L. acidophilus* per capsule, tid, 10 days	ITT 86.7% (52/60) PP 89.7% (52/58)	ITT 70.0% (42/60) PP 71.2% (42/59)	10.0% (6/60) 10.0% (6/60)
Armuzzi a[16]	2001	Italy	60 (30/30)	asymptomatic adults	UBT +ELISA/UBT (6 weeks later)	rabeprazole 20 mg bid, clarithromycin 500 mg bid, tinidazole 500 mg bid, 7 days	a probiotic preparation containing 6×10∧9 *Lactobacillus GG* (Giflorex, Errekappa Euroterapici S.p.A, Milan, Italy), bid, 14 days	ITT 83.3% (25/30)	ITT 80.0% (24/30)	40.0% (12/30) 33.3% (10/30)
Armuzzi b 17]	2001	Italy	120 (60/60)	symptomatic adults	UBT +ELISA/UBT (6 weeks later)	pantoprazole 40 mg bid, clarithromycin 500 mg bid, tinidazole 500 mg bid, 7 days	a probiotic preparation containing 6×10∧9 *Lactobacillus GG* (Giflorex, Errekappa Euroterapici S.p.A, Milan, Italy), bid, 14 days	ITT 80.0% (48/60) PP 80.0% (48/60)	ITT 70.0% (42/60) PP 80.7% (46/57)	43.3% (26/60) 61.7% (37/60)
Cremonini LB[18]	2002	Italy	42 (21/21)	asymptomatic adults	UBT/UBT (5–7 weeks later)	rabeprazole 20 mg bid, clarithromycin 500 mg bid, tinidazole 500 mg bid, 7 days	a probiotic preparation containing 6×10∧9 *Lactobacillus GG* (Giflorex, Errekappa Euroterapici, Milan, Italy), bid, 14 days	ITT 76.2% (16/21) PP 76.2% (16/21)	ITT 76.2% (16/21) PP 80.0% (16/20)	14.3% (3/21) 57.1% (12/20)
Cremonini SM[18]	2002	Italy	42 (22/21)	asymptomatic adults	UBT/UBT (5–7 weeks later)	rabeprazole 20 mg bid, clarithromycin 500 mg bid, tinidazole 500 mg bid, 7 days	a probiotic preparation containing 5×10∧9 *Saccharomyces boulardii* (Codex, SmithKline Beecham, Milan, Italy), bid, 14 days	ITT 77.3% (17/22) PP 85.0% (17/20)	ITT 76.2% (16/21) PP 80.0% (16/20)	13.6% (3/22) (57.1% (12/20)
Cremonini MS[18]	2002	Italy	42 (21/21)	asymptomatic adults	UBT/UBT (5–7 weeks later)	rabeprazole 20 mg bid, clarithromycin 500 mg bid, tinidazole 500 mg bid, 7 days	a combination of 5×10∧9 live *Lactobacillus acidophilus* and *Biphidobacterium lactis* (Ferzym, Specchiasol, Milan, Italy), bid, 14 days	ITT 85.7% (18/21) PP 85.7% (18/21)	ITT 76.2% (16/21) PP 80.0% (16/20)	23.8% (5/21) 57.1% (12/20)
Sheu [19]	2002	China (Taiwan)	160 (80/80)	symptomatic adults	Histology+RUT/UBT (8 weeks later)	lansoprazole 30 mg bid, clarithromycin 500 mg bid, amoxicillin 1 g tid, 7 days	200 mL AB-Yogurt containing 5×10∧9 *Lactobacillus* and *Bifidobacterium* (President Corp., Tainan, Taiwan), bid, 5 weeks	ITT 91.3% (73/80) PP 94.8% (73/77)	ITT 78.8% (63/80) PP 87.5% (63/72)	/
Nista [20]	2004	Italy	106 (54/52)	asymptomatic adults	UBT/UBT (6 weeks later)	rabeprazole 20 mg bid, clarithromycin 500 mg bid, amoxicillin 1 g bid, 7 days	a probiotic preparation containing 2×10∧9 *B. clausii* (Enterogermina, Sanofi–Synthelabo OTC, Milan, Italy), tid, 14 days	ITT 72.2% (39/54) PP 78.0% (39/50)	ITT 71.2% (37/52) PP 74.0% (37/50)	74.0% (40/54) 82.7% (43/50)
Myllyuom [21]	2005	Finland	47 (23/24)	asymptomatic adults	UBT +ELISA/UBT+ELISA (6 weeks later)	lansoprazole 30 mg bid, clarithromycin 500 mg bid, amoxicillin 1 g tid, 7 days	65 mL milk-based fruit drink containing 1×10∧9 cfu/mL of *Lactobacillus GG*, *L. rhamnosus LC705*, *Bifidobacterium breve Bb99* and *Propionibacterium freudenreichii ssp. shermanii JS* (Valio Ltd, Helsinki, Finland), bid during the eradication treatment and qd for the following 3 weeks	ITT 91.3% (21/23) PP 91.3% (21/23)	ITT 79.2% (19/24) PP 79.2% (19/24)	86.9% (20/23) 91.7% (22/24)
Sykora[22]	2005	UK	86 (39/47)	symptomatic children	Histology +RUT+ HpSA/UBT + HpSA (4 weeks later)	omeprazole 10 mg (15–30 kg) or 20 mg (30 kg) bid, clarithromycin 7.5 mg/kg bid, amoxicillin 25 mg/kg bid, 7 days	100 mL of fermented milk containing 10∧10 cfu/mL *L. casei DN-114001*, qd, 14 days	ITT 84.6% (33/39) PP 91.7% (33/36)	ITT 57.4% (27/47) PP 79.2% (27/44)	23.1% (9/39) 21.3% (10/47)
Goldman[23]	2006	Argentina	65 (33/32)	symptomatic children	UBT/UBT (4 weeks later)	omeprazole 1 mg/kg qd, clarithromycin 15 mg/kg qd, amoxicillin 50 mg/kg qd, 7 days	250 mL commercial yogurt containing 10∧7 cfu/mL *Bifidobacterium animalis* and *Lactobacillus casei*	ITT 45.5% (15/33) PP 45.5% (15/33)	ITT 37.5% (12/32) PP 37.5% (12/32)	/
Lionetti[24]	2006	Italy	40 (20/20)	symptomatic children	Histology +RUT+ UBT/UBT (20 days later)	omeprazole 1 mg/kg/d plus amoxycillin 50 mg/kg/d for 5 days; omeprazole 1 mg/kg/d, clarithromycin 15 mg/kg/d and tinidazole 20 mg/kg/d for the next 5 days	each pill containing 10∧8 cfu of *L. reuteri ATCC 55730* (SD2112) (BioGaia, Sweden), qd, 20 days	ITT 85.0% (17/20) PP 89.5% (17/19)	ITT 80.0% (16/20) PP 84.2% (16/19)	/
Zieminak[25]	2006	Poland	245 (53/192)	symptomatic adults	UBT/UBT (6 weeks later)	pantoprazole 40 mg bid, clarithromycin 500 mg bid, amoxicillin 1 g bid, 10 days	Lacidofil containing *Lactobacillus acidophilus* and *Lactobacillus rhamnosus*, bid, 10 days	ITT 96.2% (51/53) PP 96.2% (51/53)	ITT 85.9% (165/192) PP 85.9% (165/192)	/
Cindoruk[26]	2007	Turkey	124 (62/62)	symptomatic adults	Histology/UBT (6 weeks later)	lansoprazole 30 mg bid, clarithromycin 500 mg bid, amoxicillin 1 g bid, 14 days	250 mg sachets of *Saccharomyces boulardii* (Reflor, Sanofi-Synthelabo Ilac A.S., Istanbul, Turkey), bid, 14 days	ITT 71.0% (44/62) PP 71.0% (44/62)	ITT 59.7% (37/62) PP 59.7% (37/62)	22.6% (14/62) 59.7% (37/62)
De Bortoli N [27]	2007	Italy	206 (105/101)	symptomatic adults	HpSA+ UBT/UBT (8 weeks later)	esomeprazole 20 mg bid, clarithromycin 500 mg bid, amoxicillin 1 g bid, 7 days	bLf 20 mg bid; Pb containing 5×10∧9 *Lactobacillus plantarum*, 2×10∧9 L. reuterii, 2×10∧9 *L. caseisubsp*.*rhamnosus*, 2×10∧9 *Bifidobacterium infantis* and *B.longum*, 1×10∧9 L. *salivarius*, 1×10∧9 *L. acidophilus*, 5×10∧9 *Streptococcus termophilus*, and 1×10∧9 L. *sporogenes*, bid, 7 days	ITT 88.6% (93/105) PP 92.1% (93/101)	ITT 72.3% (73/101) PP 76.0% (73/96)	9.5% (10/105) 40.6% (41/101)
Francavill [28]	2008	Italy	40 (20/20)	symptomatic adults	Histology+HpSA+ UBT/HpSA+ UBT (4 weeks later)	rabeprazole 20 mg bid plus amoxicillin 1 g, bid for 5 days; rabeprazole 20 mg bid, clarithromycin 500 mg bid and tinidazole 500 mg bid for the next 5 days	each tablet containing 10∧8 *L. reuteri* ATCC 55730 (Reuterin, Nóos), qd, 4 weeks	ITT 85.0% (17/20) PP 85.0% (17/20)	ITT 80.0% (16/20) PP 80.0% (16/20)	/
Kim[29]	2008	Korea	347 (168/179)	symptomatic adults	Histology+UBT/UBT (4 weeks later)	standard PPI bid, clarithromycin 500 mg bid, amoxicillin 1 g bid, 7 days	150 mL Will yogurt containing *L. acidophilus* HY 2177 (> 10∧5 cfu/mL), *L. casei* HY 2743 (> 10∧5 cfu/mL), *B. longum* HY 8001 (> 10∧6 cfu/mL), and *S. thermophilus* B-1 (> 10∧8 cfu/mL) (Korea Yakult Company Limited; Chunan-Si, Chungnam, S. Korea), qd, 3 weeks	ITT 79.2% (133/168) PP 87.5% (133/152)	ITT 72.1% (129/179) PP 78.6% (129/164)	41.1% (69/168) 26.3% (47/179)
Scaccianoce LB[30]	2008	Italy	33 (17/16)	symptomatic adults	Histology/UBT (4–6 weeks later)	lansoprazole 30 mg bid, clarithromycin 500 mg bid, amoxicillin 1 g bid, 7 days	each tablet containing 10∧8 *L. reuteri* ATCC 55730 (Reuterin, Nóos), bid, 7 days	ITT 52.9% (9/17) PP 52.9% (9/17)	ITT 62.5% (10/16) PP 66.7% (10/15)	5.9% (1/17) 25.0% (4/16)
Scaccianoce MS[30]	2008	Italy	31 (15/16)	symptomatic adults	Histology/UBT (4–6 weeks later)	lansoprazole 30 mg bid, clarithromycin 500 mg bid, amoxicillin 1 g bid, 7 days	a probiotic mixture with *Lactobacillus plantarum* (5×10∧9), *L. reuteri* (2×10∧9), *Lactobacillus casei subsp*. *Rhamnosus* (2×10∧9), *Bifidobacterium infantis* (2×10∧9), *Bifidobacterium longum* (2×10∧9), *Lactobacillus salivarius* (1×10∧9), *Lactobacillus acidophilus* (1×10∧9), *Streptococcus termophilus* (5×10∧9), and *Lactobacillus sporogenes* (1×10∧9); 5 g/dose, bid, 7 days	ITT 53.3% (8/15) PP 53.3% (8/15)	ITT 62.5% (10/16) PP 66.7% (10/15)	20.0% (3/15) 25.0% (4/16)
Hurduc[31]	2009	Romania	90 (48/42)	symptomatic children	Histology+RUT/Histology+RUT (4–6 weeks later)	omeprozole or esomeprazole 0.5 mg/kg bid, clarothromycin 7.5 mg/kg bid, amoxicillin 25 mg/kg bid, 7–10 days	*Saccharomyces boulardii* (Enterol; Biocodex, Gentilly Cedex, France), 250 mg/day, bid, 4 weeks	ITT 93.8% (45/48) PP 100% (45/45)	ITT 80.9% (34/42) PP 75.6% (34/42)	8.3% (4/48) 30.9% (13/42)
Szajewsk[32]	2009	Poland	66 (34/32)	asymptomatic children	Histology+ RUT+UBT/UBT (4–6 weeks later)	omeprozole 0.5 mg/kg bid, clarithromycin 10 mg/kg bid, amoxicillin 25 mg/kg bid, 7 days	10∧9 cfu/mL *Lactobacillus GG*, bid, 7 days	ITT 67.6% (23/34) PP 67.6% (23/34)	ITT 68.8% (22/32) PP 68.8% (22/32)	52.9% (18/34) 40.6% (13/32)
Song[33]	2010	Korea	661 (330/331)	symptomatic adults	UBT/UBT(4 weeks later)	omeprazole 20 mg bid, clarithromycin 500 mg bid, amoxicillin 1 g bid, 7 days	3×10∧9 cfu/g *Saccharomyces boulardii* (Bioflor250, Kuhnil Pharmacy, Seoul, Korea), tid, 4 weeks	ITT 80.0% (264/330) PP 85.4% (264/309)	ITT 71.6% (237/331) PP 80.0% (237/296)	/
Yasar[34]	2010	Turkey	76 (38/38)	symptomatic children	Histology/UBT (4 weeks later)	pantoprazole 40 mg bid, clarithromycin 500 mg bid, amoxicillin 1 g bid, 14 days	125 ml yogurt containing 10∧10 cfu/g *Bifidobacterium* DN-173 010, qd, 14 d	ITT 65.8% (25/38) PP 65.8% (25/38)	ITT 52.6% (20/38) PP 52.6% (20/38)	/
Bekar[35]	2011	Turkey	82 (46/36)	symptomatic adults	UBT/UBT (45 days later)	lansoprazole 30 mg bid, clarithromycin 500 mg bid, amoxicillin 1 g bid, 14 days	250 mL kefir, bid, 141d	ITT 78.3% (36/46) PP 78.3% (36/46)	ITT 50.0% (18/36) PP 50.0% (18/36)	/
Madeiros[36]	2011	France	62 (31/31)	asymptomatic adults	Histology+ culture/UBT (≥6 weeks later)	esomeprazole 20 mg bid, clarithromycin 500 mg bid, amoxicillin 1 g bid, 8 days	Lacteol (BioSaúde laboratories, Portugal), capsule containing 5×10∧9 *L. acidophilus*, bid, 8 days	ITT 83.9% (26/31) PP 83.9% (26/31)	ITT 77.4% (24/31) PP 77.4% (24/31)	/
Ozdil[37]	2011	Turkey	193 (98/95)	symptomatic adults	Histology/HpSA (5 weeks later)	lansoprazole 30 mg bid, clarithromycin 500 mg bid, amoxicillin 1 g bid, 14 days	250 mg capsules containing *saccharomyces boulardii*, qd, 14 days	ITT 72.4% (71/98) PP 77.2% (71/92)	ITT 86.3% (82/95) PP 89.1% (82/92)	/
Deguchi[38]	2012	Japan	229 (115/114)	symptomatic adults	Histology-RUT-culture/UBT+HpSA (8 weeks later)	rabeprazole 10 mg bid, clarithromycin 200 mg bid, amoxicillin 750 mg bid, 7 days	yogurt containing 112 g *L. gasseri* OLL2716 (>10 cfu/g), bid, 4 weeks	ITT 82.6% (95/115) PP 89.6% (95/106)	ITT 69.3% (79/114) PP 71.2% (79/111)	5.2% (6/115) 3.5% (4/114)
Du YQ[39]	2012	China	157 (78/79)	symptomatic adults	Histology+RUT+UBT/UBT (4 weeks later)	omeprazole 20 mg bid, clarithromycin 500 mg bid, amoxicillin 1 g bid, 7 days	probiotics, containing 3×10∧7 *Lactobacillus acidophilus*, qd, 14 d	ITT 79.5% (62/78) PP 81.6% (62/76)	ITT 60.7% (48/79) PP 61.5% (48/78)	1.3% (1/78) 0.0% (0/79)
Manfredi[40]	2012	Italy	149 (73/76)	symptomatic adults	Histology+ HpSA/HpSA (8–10 weeks later)	esomeprazole 20 mg bid plus amoxicillin 1 g, bid for 5 days; esomeprazole 20 mg bid, clarithromycin 500 mg bid and tinidazole 500 mg bid for the next 5 days	Lactogermine plus (Humana Italia s.p.a., Milano, Italy), containing 10∧9 *Lactobacillus acidophilus*, 5×10∧8 *Bifidobacterium bifidum*, 10∧9 *Streptococcus thermophilus*, 10∧9 *Lactobacillus bulgaricus*	ITT 91.8% (67/73) PP 94.4% (67/71)	ITT 85.5% (65/76) PP 92.8% (65/70)	39.7% (29/73) 65.8% (50/76)
Mirzaee[41]	2012	Iran	68 (34/34)	symptomatic adults	UBT/UBT (4 weeks later)	pantoprazole 40 mg qd, clarithromycin 500 mg bid, amoxicillin 1 g bid, 7 days	150 mg probiotic yogurt, bid, 7 days	ITT 55.9% (19/34) PP 61.3% (19/31)	ITT 58.8% (20/34) PP 64.5% (20/31)	64.7% (22/34) 67.6% (23/34)
Tolone[42]	2012	Italy	68 (34/34)	symptomatic children	Histology+ UBT/UBT (4 weeks later)	omeprazole 1 mg/kg qd, amoxicillin 50 mg/kg bid, clarithromycin 15 mg/kg bid, 7 days	the PB supplement containing 5×10∧9 *Lactobacillus plantarum*, 2×10∧9 *L. reuterii*, 2×10∧9 *L.casei subsp*. *rhamnosus*, 2×10∧9 *Bifidobacterium infantis* and *B. longum*, 1×10∧9 *L. salivarius*, 1×10∧9 *L. acidophilus*, 5×10∧9 *Streptococcus termophilus*, and 1×10∧9 *L. sporogenes*, qd, 7 days	ITT 88.2% (30/34) PP 88.2% (30/34)	ITT 76.5% (26/34) PP 76.5% (26/34)	/
Ahmad[43]	2013	Iran	66 (33/33)	symptomatic children	Histology+RUT/HpSA (4–8 weeks later)	omeprazole 0.5 mg/kg bid, amoxicillin 25 mg/kg bid, furazolidone 3 mg/kg bid, 7 days	probiotic combination (restore, 10∧9 cfu/each sachet, Protexin Co, UK) containing *Lactobacillus acidophilus*, *Lactobacillus rhamnosus*, *Lactobacillus bulgaricus*, *Lactobacillus casei*, *Streptococcus thermophilus*, *Bifidobacterium infantis* and *Bifidobacterium breve*, qd, 7 days	ITT 90.9% (30/33) PP 90.9% (30/33)	ITT 69.7% (23/33) PP 69.7% (23/33)	/
Navarro[44]	2013	Brazil	107 (55/52)	symptomatic adults	Histology+RUT+UBT/UBT (8 weeks later)	lansoprazole 30 mg bid, tetracycline 500 mg bid, furazolidone 200 mg bid, 7 days	the probiotic compound containing 1.25×10∧9 CFUs *Lactobacillus acidophilus*, 1.25×10∧9 CFUs *Lactobacillus rhamnosus*, 1.25×10∧9 CFUs *Bifidobacterium bifidum* and 1.25×10∧9 CFUs *Streptococcus faecium*, bid, 30 days	ITT 89.1% (49/55) PP 96.1% (49/51)	ITT 84.6% (44/52) PP 89.8% (44/49)	58.2% (32/55) 71.2% (37/52)
Shavakhi[45]	2013	Iran	180 (90/90)	symptomatic adults	Histology+ RUT/UBT (4 weeks later)	omeprazole 20 mg bid, bismuth subcitrate 240 mg bid, amoxicillin 1000 mg bid, clarithromycin 500 mg bid, 14 days	Balance capsue containing *Lactobacillus* strains (*L. casei, L. rhamnosus, L. acidophilus*, and *L. bulgaricus*), *Bifidobacterium* strains (*B. breve* and *B. longum*), and *Streptococcus thermophiles*, total viable count is 10∧8 CFU/per capsule, bid, 14 days	ITT 76.7% (69/90) PP 82.1% (69/84)	ITT 81.1% (73/90) PP 84.9% (73/86)	18.9% (17/90) 16.7% (15/90)
Dajani[46]	2013	United Arab Emirates	206 (100/106)	symptomatic adults	Histology+RUT+UBT+HpSA/UBT (6–8 weeks later)	omeprazole 20 mg bid, clarithromycin 500 mg bid, amoxicillin 1 g bid, 7 days	3×10∧9 *B. infantis* 2036, bid, 7 days	ITT 83% (83/100) PP 83% (83/100)	ITT 68.9% (73/106) PP 68.9% (73/106)	/
Wang YH[47]	2013	China	100 (49/51)	symptomatic children	UBT/UBT (6 weeks later)	PPI 0.6–0.8 mg/kg bid, clarithromycin 10–15 mg/kg bid, amoxicillin 30–50 mg/kg bid, and metronidazole 15–20 mg/kg bid for penicillin allergic children, 14 days	each packs of probiotics containing 2 g *L. acidophilus-5* (4.7×10∧9 cfu/100 g) and 2 g *B. bifidum-12* (4.3×10∧8 cfu/100 g), <5 years old, qd; >5 years old, bid, 14 days	ITT 73.5% (36/49) PP 83.7% (26/43)	ITT 56.9% (29/51) PP 64.4% (29/45)	10.2% (5/49) 23.5% (12/51)

### Trial Quality Assessment

**Figure 2:**
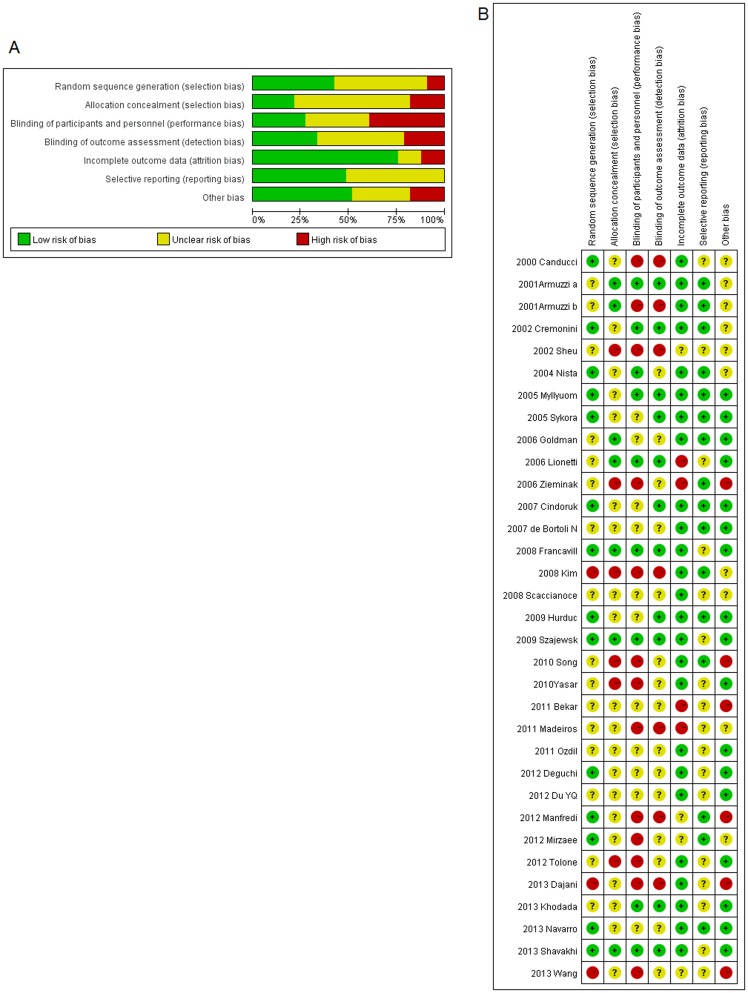
Risk of bias graph (A): review authors' judgements about each risk of bias item presented as percentages across all included studies. (B) Risk of bias summary: review authors' judgements about each risk of bias item for each included study. (+)  =  low risk of bias, (?)  =  unclear, (-)  =  high risk of bias.

Figure 2A shows authors' judgements for each Cochrane risk of bias item and Figure 2B presents the Cochrane risk of bias score for each citation included.

### Eradication Rates

**Figure 3:**
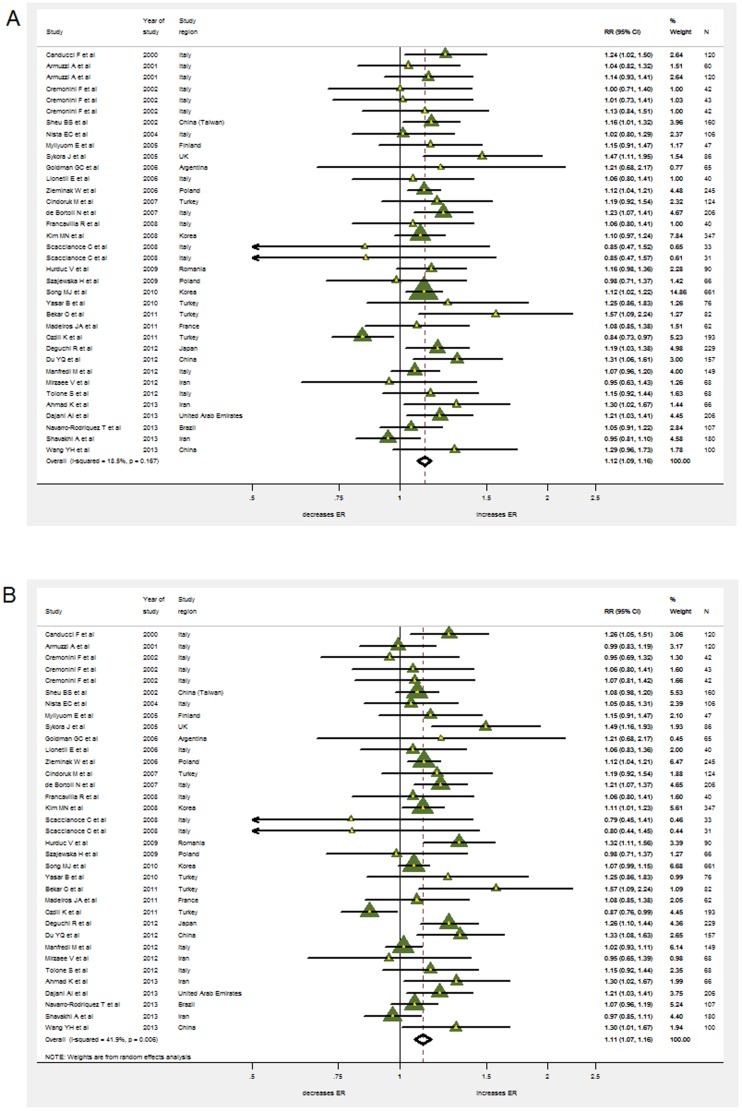
The effect of probiotics supplementation vs. without probiotics on eradication rates by intention-to-treat analysis in (A). RR, risk ratio; CI, confidence interval. The triangles represent individual studies and the size of the triangle represents the weight given to each study in the meta-analysis. The diamond represents the combined results. (B) The effect of probiotics supplementation vs. without probiotics on eradication rates by pre-protocol analysis. RR, risk ratio; CI, confidence interval. The triangles represent individual studies and the size of the triangle represents the weight given to each study in the meta-analysis. The diamond represents the combined results.

Eradication risk ratios (RRs) were available for 4459 patients (2189 in the probiotics supplementation group and 2270 in the control group). Heterogeneity was found to be low for the overall incidence of eradication rates in ITT analysis (χ^2^ = 42.97, p = 0.167, I^2^ = 18.5%) but higher for PP analysis (χ^2^ = 58.55, p = 0.006, I^2^ = 41.9%). Therefore, a fixed effects model based on Mantel-Haenszel's estimation method was used in the case of ITT and a random effects model in the case of PP analysis. Nonetheless, the pooled RRs from intention-to-treat (ITT) and from pre-protocol (PP) analysis for the probiotics supplementation group over controls were very similar with 1.122 (95% CI 1.086–1.159) and 1.114 (95% CI 1.070–1.159) respectively (Figure 3A&B).

Omitting individual studies from the meta-analysis did not change occurrence of RRs being significantly higher than 1. Funnel-plot and Harbord's modified test showed no evidence for publication bias (p = 0.64). Neither the age of the population (pediatric vs. adult, p = 0.127) nor symptom status (p = 0.314) played a role according to the meta-regression.

Studies used different strains of probiotics with 13 applying *Lactobacillus* [17]–[20], [24], [26], [27], [30], [32], [34], [38], [40], [41], two using *Bifidobacterium* [36], [48], one using *Bacillus clausii* [22], five using *Saccharomyces* [20], [28], [33], [35], [39] and 15 using multistrain [20], [21], [23], [26], [29], [31], [32], [37], [42]–[47], [49]. Among those studies applying individual probiotic supplementation, four specific species were effective: *Lactobacillus acidophilus* (pooled RR = 1.235, 95% CI 1.090–1.400), *Lactobacillus casei* DN-114001 (pooled RR = 1.473, 95% CI 1.13–1.949), *Lactobacillus gasseri* (pooled RR = 1.192, 95% CI 1.028–1.382), *Bifidobacterium infantis* 2036 (pooled RR = 1.205, 95% CI 1.031–1.408). While double-blinded (pooled RR = 1.118, 1.045–1.196) and non-blinded trials (pooled RR = 1.120, 95% CI 1.080–1.162) basically arrived at the same results, sub-group analysis by effectiveness of the antibiotic therapy in the control group revealed that supplementation with probiotics solely increased eradication rates in relatively ineffective therapies.

**Figure 4:**
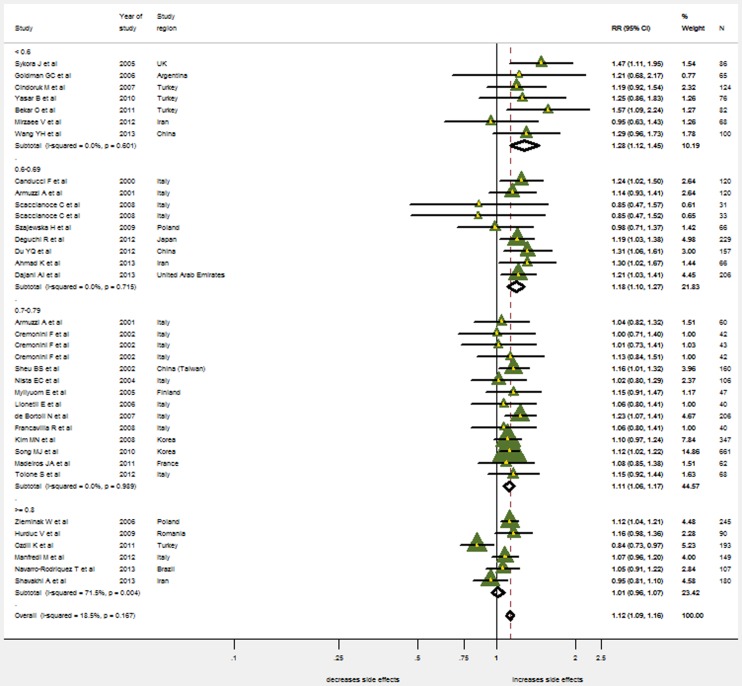
Meta-analysis of eradication rates by eradication rates due to antibiotic therapy only (control group). RR, risk ratio; CI, confidence interval. The triangles represent individual studies and the size of the triangle represents the weight given to each study in the meta-analysis. The diamond represents the combined results.

Another sub-group analysis was done according to the effectiveness of eradication regimens. The less effective antibiotic therapies were, the more useful probiotic supplementation was: when eradication rate <60%, pooled RR = 1.28, 1.12–1.45; when eradication rate within 60%–69%, pooled RR = 1.18, 1.10–1.27; when eradication rate within 70%–79%, pooled RR = 1.11, 1.06–1.17; while if eradication rate over 80%, the supplementation was useless (pooled RR = 1.01, 0.96–1.77). (Figure 4)

### Side Effects

**Figure 5:**
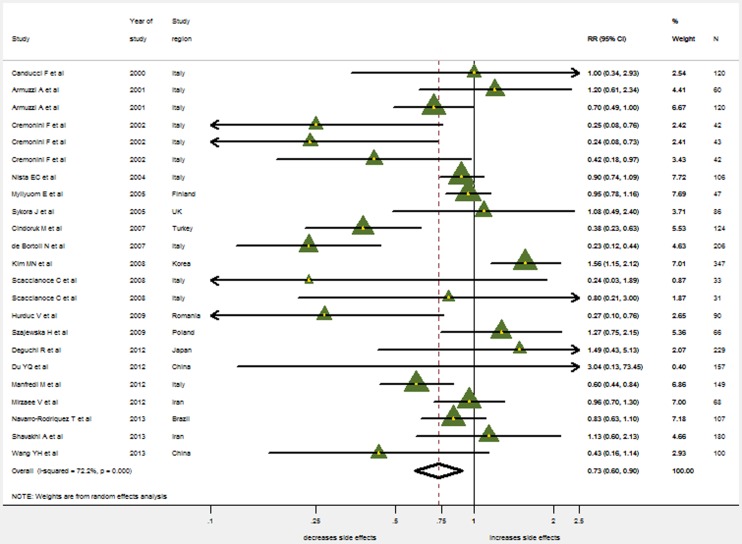
The effect of probiotics supplementation vs. without probiotics on total side effects. RR, risk ratio; CI, confidence interval. The triangles represent individual studies and the size of the triangle represents the weight given to each study in the meta-analysis. The diamond represents the combined results.

**Figure 6:**
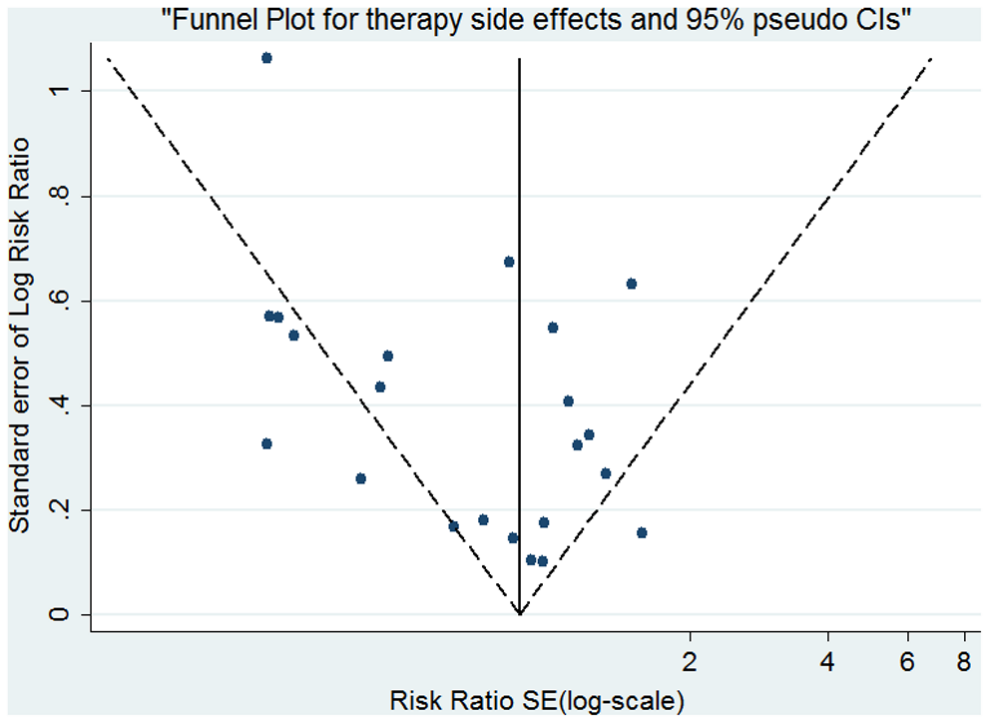
Funnel plot analysis of publication bias for side effects.

There were 20 trials (2487 patients, 1269 in the probiotics supplementation group and 1218 in the control group) which provided data on the overall incidence of side effects. Significant heterogeneity was found for the overall occurrence of side effects (I^2^ = 72.2%, P<0.001). Therefore, the random effects model was used. The pooled RR in the probiotics supplementation over control was 0.735 (95% CI 0.598–0.902) (Figure 5). While Harbord's modified test for publication bias was insignificant (p = 0.17), the Funnel Plot did reveal some asymmetry (Figure 6): Small studies showing a considerable reduction in side-effects occur more often than those only showing a small reduction. When stratified by probiotics, only the pooled RR for *Saccharomyces boulardii* indicates a significant reduction in side effects (0.335, 95% CI 0.220–0.510; LB: 0.892, 95% CI 0.632–1.259; MS: 0.760, 95% CI 0.568–1.017; BB: 0.895: 0.737–1.087).

**Figure 7:**
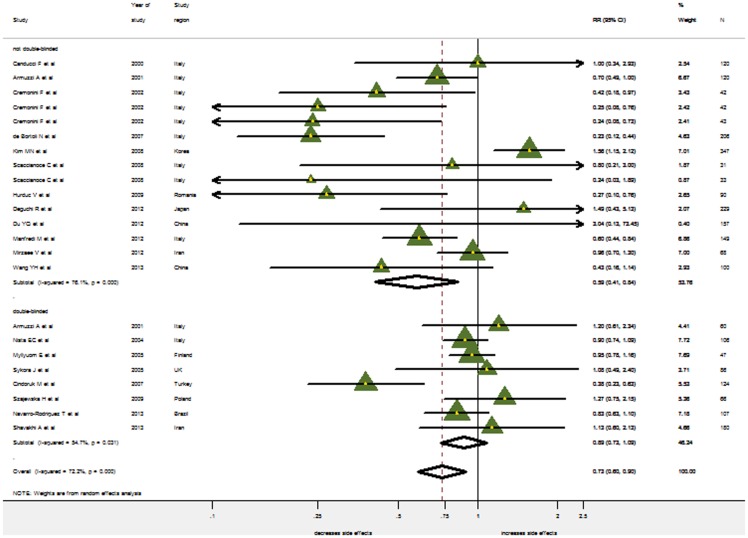
Meta-analysis of side effects by double-blinded and nondouble-blinded trials. RR, risk ratio; CI, confidence interval. The triangles represent individual studies and the size of the triangle represents the weight given to each study in the meta-analysis. The diamond represents the combined results.

Only eight studies reporting the overall incidence of side effects were blinded and solely results from non-blinded trials provided evidence for the reduction of side-effects (pooled RR = 0.589, 95% CI: 0.412–0.842), while double blinded studies did not (pooled RR = 0.889, 95% CI: 0.728–1.085) (Figure 7).

## Discussion

This is the largest meta-analysis on the effects of probiotics on *H. pylori* eradication and side effects conducted to date. The quality of the trials was medium to low and no study had a low risk of bias across all Cochrane criteria. Overall, this analysis of 33 RCTs suggests that supplementation of antibiotic therapy with probiotics increases eradication rates compared to a placebo or no intervention. However, regarding individual probiotic strains, this could only be confirmed for several strains of *Lactobacilus* and one strain of *Bifidobacterium*. Moreover, probiotics only demonstrated additional effects if the eradication rates in the control groups were relatively low, i.e. cases where the antibiotic therapy was relatively ineffective. Although, we found an overall decrease of total side effects, this held true only for *Saccharomyces boulardii* and non-blinded RCTs.

In comparison with previously published meta-analysis [8], [9], [10], [11], [12], [13], [14], our updated study represents the most comprehensive analysis. Various potential influence factors were taken into consideration such as age, symptom status, eradication therapy regime, eradication rate, bacterial strain and blinding method. Moreover, from the previous studies, six [8], [10], [11], [12], [13], excluded trials using Quadruple therapy as the co-intervention and none performed subgroup analysis with regard to the efficacy of antibiotic therapy. Moreover, sensitivity analysis for side effects had not been conducted so far. Our results confirm previous meta-analyses only to some extent. At a first glance, probiotics seem effective in increasing eradication rates and decreasing side effects. At a closer look, however, evidence only supports these claims for specific probiotic strains, ineffective antibiotic therapies and low-quality trials (i.e. non-blinded studies) as far as side effects are concerned.

Before Marshall found *H. pylori* in 1984 [57], the stomach was considered to be a sterile organ due to its low pH level. In the aftermath, ‘ *H. pylori* has been intensively studied and recent sequencing analysis of other gastric microbiota shows that *H. pylori* is not alone’ [58]. Although there are only 10∧2-4 cfu/g in the gastric mucosa [59], these commensal bacteria can play an important role in maintaining human health. Probiotic supplementation as an approach to manipulate gastrointestinal flora has been intensively studied.

While all previously conducted meta-analysis concluded that supplementation of antibiotic therapy with probiotics is effective in increasing eradication rates [8], [9], [10], [11], [12], our study identified only four effective individual strain: *Lactobacillus acidophilus* (Pubmed ID: 190198), *Lactobacillus casei* DN-114001 (Pubmed ID: 35628), *Lactobacillus gasseri* (Pubmed ID: 35528), *Bifidobacterium infantis* 2036 (Pubmed ID: 41468). However, more than one trial was conducted only for *Lactobacillus acidophilus* so far, limiting the generalizability of the findings regarding the other three strains. While *Lactobacillus GG* had previously been reported to be an effective strain [8], we do reach the same conclusion here.

Moreover, our results suggest that probiotic supplementation is only useful in less effective (eradication rate <80%) antibiotic therapies. Acceptable success rates have often been defined as 80% or more on an ITT basis [2]. In those effective regimens, probiotic supplementation may not be needed. On the other hand, however, increasing resistance to antibiotics, for example clarithromycin, has been noted [2]. Probiotics supplementation may be a potential approach for eliminating resistant strains. However, no study included in this meta-analysis reported the detection of antibiotic resistance. Further research is warranted to clarify this issue.

The occurrence of adverse effects is one of the major drawbacks of antibiotic treatment. Although antibiotics may modify the composition of intestinal bacteria, broad spectrum antibiotics also often lead to gastrointestinal side effects [20], [60]. Results regarding the effectiveness of probiotics in reducing side effects from previous meta-analyses have been inconclusive [8], [9], [10], [11], [12]. While we found that probiotics reduce the overall occurrence of side effects in the pooled data, this result must be taken with caution. Only one specific strain significantly reduced side effects and this overall result was only confirmed for non-blinded trials. While eradication rates can be determined with objective measures, assessment of side effects must rely on patients' subjective reporting. When patients are not blinded, their reports are likely to be biased due to awareness of a respective intervention.

Limitations of this meta-analysis include a limited number of high quality trials that could be analyzed, particularly regarding individual probiotic strains and side effects. Only one controlled trial was conducted for the following specific species *Bacillus clausii*, *Bifidobacterium* DN-173 010, *Lactobacillus casei* DN-114001, *Lactobacillus gasseri*, *Bifidobacterium infantis* 2036. Accordingly, results regarding those strains need to be interpreted with caution. Thirteen of the analyzed trials did not provide data on overall occurrence of side effects. Moreover, tools used for measuring side effects and reporting individual side effects largely differed across studies. From 20 studies reporting on the overall incidence of side effects, only eight were blinded.

## Conclusions

The pooled data suggests that supplementation with specific strains of probiotics compared with eradication therapy may be considered as an option for increasing eradication rates of *H. pylori*, particularly when antibiotic therapies are relatively ineffective.

## Supporting Information

Checklist S1 **PRISMA Checklist.**
(DOC)
